# Vascular Leakage in Dengue Hemorrhagic Fever Is Associated with Dengue Infected Monocytes, Monocyte Activation/Exhaustion, and Cytokines Production

**DOI:** 10.1155/2015/917143

**Published:** 2015-02-05

**Authors:** Sirichan Chunhakan, Punnee Butthep, Sutee Yoksan, Kanchana Tangnararatchakit, Ampaiwan Chuansumrit

**Affiliations:** ^1^Department of Pathology, Faculty of Medicine Ramathibodi Hospital, Mahidol University, Bangkok 10400, Thailand; ^2^Vaccine Development Center, Mahidol University, Salaya Campus, Nakhon Pathom, Thailand; ^3^Department of Pediatrics, Faculty of Medicine Ramathibodi Hospital, Mahidol University, Bangkok 10400, Thailand

## Abstract

The vascular leakage was shown by the increment of hematocrit (Hct), dengue viral infected monocyte, monocyte status, and cytokines production in patients infected with dengue virus. Dengue viral antigens were demonstrated in monocytes (CD14+) from peripheral blood mononuclear cells. The increased levels of Hct, interleukin- (IL-) 10, and tumor necrosis factor-alpha (TNF-*α*) were detected in dengue fever (DF), dengue hemorrhagic fever (DHF) and dengue shock syndrome (DSS) patients as compared with other febrile illnesses (OFIs). The highest levels of Hct and IL-10 were detected in DSS patients as compared with other groups (*P* < 0.05) especially on one day before and after defervescence. The unstimulated and lipopolysaccharide- (LPS-) stimulated monocytes from DSS patients showed the significantly decreased of intracellular IL-1*β* and TNF-*α*. In addition, the lowest level of mean fluorescence intensity (MFI) of CD11b expression on monocytes surface in DSS patients was also demonstrated. Furthermore, the negative correlations between IL-10 levels and intracellular IL-1*β* and MFI of CD11b expression in unstimulated and LPS-stimulated monocytes were also detected. Nevertheless, not only were the relationships between the prominent IL-10 and the suppression of intracellular monocyte secretion, namely, IL-1*β*, TNF-*α*, demonstrated but also the effect of vascular leakage was observed.

## 1. Introduction

The complication of DHF/DSS is thought to result from a complex interplay between the virus, host genetics, and host immune factors also depending on individual, epidemiologic, and ecologic conditions [1, 2]. Several studies reported that monocytes are natural host cells for dengue virus [3, 4]. Monocytes have been implicated in both pathogenesis and protection of dengue such as the production of interferon-*α* in response to dengue virus [5]. On the other hand, monocytes promote dengue pathogenesis by being the primary vessel of virus propagation [6]. In addition, monocytes/macrophage can produce cytokines and chemokines that compromise the integrity of the endothelial cell layer [7–9], possibly leading to vascular leakage, the hallmark of severe dengue diseases [10, 11]. Furthermore, monocytes are known to die spontaneously by apoptosis and this can be prevented by appropriate stimuli such as LPS, TNF-*α*, and IL-1*β* [12, 13]. Dengue-infected monocytes could stimulate cytokines/chemokines production such as TNF-*α* and IL-1*β* which are known to activate vascular endothelial cells and lead to vascular leakage.

IL-10 is a major anti-inflammatory cytokine that has been associated with several diseases and is considered as an important immunoregulatory mediator produced by monocytes, dendritic cells, and T and B lymphocytes [14]. Recently, an elevated level of IL-10 has been reported in patients infected with dengue virus, especially in severe dengue infection [15, 16]. Both TNF-*α* and IL-10 have been involved in the thrombocytopenia and hemorrhagic manifestation observed during dengue infection [17]. Previously, there are many studies which reported that IL-10 is critically involved in the genesis of DHF, an increase in IL-10 levels has been correlated with platelet decay in dengue infection, and IL-10 may be downregulating lymphocyte and platelet function [18–20]. In addition, IL-10 could be involved in the induction of T cell apoptosis described in the secondary virus infection [21]. The infected monocytes, or memory T cells activated by infected monocytes as antigen presenting cells, could be the main sources of IL-10 in dengue infections [22]. Recently, the detection of dengue viral antigen in peripheral blood mononuclear cells which can provide a rapid diagnosis of dengue virus infection and the alteration of cytokines and chemokines in the febrile episode which related to DSS patients were reported [23, 24]. Therefore, the aims of this study are to demonstrate the hematological changes especially the increment of Hct which indicate the vascular leakage, dengue viral infected monocytes, and the monocyte's markers of activation/exhaustion as shown by the proinflammatory and anti-inflammatory cytokines production including of TNF-*α*, IL-1*β*, and IL-10 especially intracellular cytokines in monocytes of patients infected with dengue virus in order to understand the cause and effect relationship which may lead to the severity of the diseases.

## 2. Materials and Methods

### 2.1. Clinical Samples and Definitions

Thai children with suspected dengue virus infection admitted at the Department of Pediatrics, Faculty of Medicine Ramathibodi Hospital, Mahidol University, Bangkok, Thailand, were enrolled in this study. The subjects consisted of patients with DF, DHF grade I, DHF grade II, and DHF grades III and IV (DSS) [25]. Diagnosis of dengue infection was confirmed by viral isolation using virus inoculation technique [26] and/or presence in acute and convalescent sera of dengue-specific IgM [27] and IgG [28] determined by ELISA method. Other febrile illness (OFIs) patients, having no dengue virus-specific IgM and IgG responses and negative for isolated dengue virus, were included as controls. Ethical approval was obtained from the Committee on Human Rights Related to Researches involving Human Subjects of Faculty of Medicine Ramathibodi Hospital, Mahidol University, Bangkok, Thailand. Informed consent was obtained from parents or caregivers.

Day 0 was designated as day of defervescence, when temperature dropped below 37.5°C without a subsequent elevation. Days prior to defervescence were designated as Day −1, Day −2, and so on, and days after defervescence were designated as Day +1, Day +2, and so on. The complete blood count was performed in all samples using automated blood cell analyzer (Sysmex XE-5000, Sysmex, Japan).

### 2.2. Identification of Monocyte as a Target Cell of Dengue Virus Infection

The smear of peripheral blood mononuclear cells (PBMCs) was prepared by the technique as previously described [29]. The smear was doubled-stained with fluorescein isothiocyanate- (FITC-) conjugated polyvalent dengue 1–4 antisera [30] and R-phycoerythrin-cyanin 5.1- (PC5-) conjugated CD14 (Immunotech, Marseille, France), a specific molecule for monocyte population, and examined under a laser scanning confocal microscope: MRC-1024 (Bio-Rad, Hertfordshire, UK). Positive control consisted of monolayer LLC-MK2 cells infected with dengue virus type 2 (16681) strain and harvested on Day 7 (provided by Vaccine Development Center, Mahidol University) and negative control was provided by white blood cells with negative staining on the smear.

### 2.3. Determination of IL-10, TNF-*α*, and IL-1*β*

The plasma levels of interleukin-10, TNF-*α*, and IL-1*β* in patients infected with dengue virus were determined by using the commercial cytokines and growth factors array I (Randox, London, UK). All samples were examined as undiluted plasma according to the manufacturer's protocol. The researchers who carried out the study were blind to the clinical status, results of viral isolation, and dengue-specific IgM and IgG.

### 2.4. Assessment of Monocyte Status Using Flow Cytometry

For intracellular cytokines determination, sodium heparinized blood was incubated with GolgiPlug: brefeldin A (BFA) (BD Biosciences, San Jose, USA) for 4 hours at 37°C in 5% CO_2_ with and without 1 *μ*g/mL lipopolysaccharide (LPS) (Sigma, St. Louis, USA). Unstimulated and LPS-stimulated blood samples were lysed with FACS Lysing solution (BD Biosciences, USA). After centrifuging and decanting the supernatant, WBC pellets were washed and stained with PC5-conjugated CD14 (Immunotech, Marseille, France) for 15 minutes and, after washing, the samples were incubated for 10 minutes with FACS permeabilizing solution (BD Biosciences, San Jose, USA) and incubated with FITC-conjugated antihuman TNF-*α* (R&D Systems, Minneapolis, USA) and phycoerythrin- (PE-) conjugated antihuman IL-1*β* (R&D Systems, Minneapolis, USA) for 30 minutes in the dark at room temperature. Then, the stained cells were washed and resuspended in 1% paraformaldehyde.

For CD11b expression on the surface of monocytes, sodium heparinized blood was lysed with 4°C FACS lysing solution (BD Biosciences, San Jose, USA) for 10 minutes. After centrifugation and discarding the supernatant, the WBC pellets were washed and incubated with PC5-conjugated CD14 and FITC-conjugated CD11b (Immunotech, Marseille, France) for 20 minutes at 4°C in the dark. The stained cells were washed and analyzed within 24 hours by FACSCalibur flow cytometer (BD Biosciences, San Jose, USA) and the isotype control antibodies (mouse IgG1 conjugated with FITC and PE) (R&D Systems, Minneapolis, USA) were used as the negative markers. The assessment of monocyte status was performed by gating on CD14-positive cells including percentage of CD14+ cells expressing TNF-*α* and IL-1*β* in both resting and activated stage for intracellular cytokines and mean fluorescence intensity (MFI) of monocyte (CD14+) with CD11b expression.

### 2.5. Statistical Analysis

The results were compared among the studied groups of patients infected with dengue virus based on clinical assessment (DF, DHF I, DHF II, and DSS) and OFIs patients. The nonparametric Mann-Whitney *U* test and the Kruskal-Wallis test were used to calculate statistical significance for the differences in markers of monocyte activation/exhaustion between groups of patients. Comparisons between results obtained at different time points were performed using the Wilcoxon signed rank test and to study the linear relationship between variables, Spearman's correlation coefficients were calculated. Differences were considered significant with *P* < 0.05.

## 3. Results

### 3.1. Demographic Information and Hematological Determinations

Clinical diagnosis and demographic information are shown in Table 1 and the distribution of studied samples followed by the day of illness related to fever is shown in Table 2. The mean of maximum, minimum, and percentage of increment of Hct in patients with DF, DHF grades I and II, DSS, and other OFIs patients were shown in Table 3. The highest level of mean increment of Hct (%) was detected in DSS patients. Mean ± SD of platelet counts in DF, DHF grades I and II, DSS, and OFIs patients was shown in Table 4. The lowest platelet numbers were demonstrated in DSS patients on Day 0, and the thrombocytopenia was shown in other types of dengue-infected patients.

### 3.2. Identification of Dengue Viral Antigen in Peripheral Blood Monocytes

**Figure 1:**
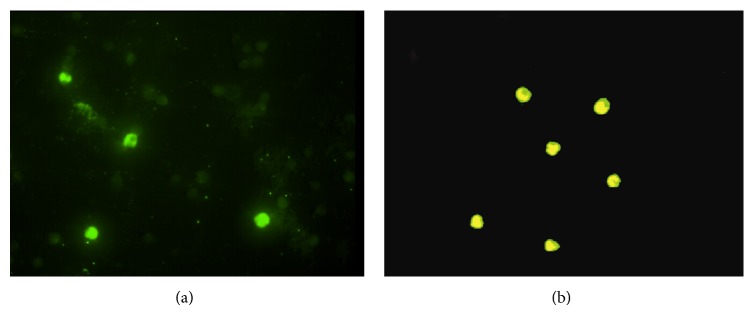
Demonstration of dengue viral antigen in monocytes of patients infected with dengue virus as dengue viral antigen positive-stained cells in panel (a) and double-stained for dengue viral antigen and CD14 positive cells in panel (b).

Dengue viral antigen found in PBMCs was demonstrated in CD14 positive cells or monocytes. The results of double stained detection were shown in Figure 1 as dengue viral antigen positive-stained cells in panel (a) and double-stained cells for dengue viral antigen and CD14 positive cells in panel (b).

### 3.3. Plasma Levels of IL-10, TNF-*α*, and IL-1*β*

#### 3.3.1. Interleukin-10 (IL-10)

The levels of IL-10 from Day −2 to Day +2 in all groups of patients were elevated as compared to the levels on the day of convalescence. The highest level was detected in DSS patients especially on Day −1 and Day +1 (*P* < 0.05), consequences were in DHF II, DHF I, and DF patients, and the lowest level was demonstrated in OFIs patients. On the day of convalescence, the levels of IL-10 in all groups were less than 1.5 pg/mL (Table 5).

#### 3.3.2. Tumor Necrosis Factor-*α* (TNF-*α*)

On the day of defervescence, the level of TNF-*α* in DSS patients was significantly lower than the levels in DF and DHF II (*P* < 0.05), whereas the highest level was shown in DSS patients on Day +1. From Day −2 to Day +2, the mean levels of TNF-*α* in all patients infected with dengue virus was significantly higher than that on convalescent day (*P* < 0.05) except in DHF I patients on Day +2 (Table 6).

#### 3.3.3. Interleukin-1*β* (IL-1*β*)

The levels of IL-1*β* in DHF patients did not show the significant difference as compared to the levels on the day of convalescence. In contrast, the increased levels of IL-1*β* were detected in DF and OFIs patients from Day −1 to Day +2 as compared with the levels on the day of convalescence. From Day −1 to Day +2, the mean levels of IL-1*β* in DF patients were higher than the other groups. In DSS patients, the mean levels of IL-1*β* on Day +1 and Day +2 were lower than those on convalescent day (Table 7).

### 3.4. Intracellular TNF-*α* and IL-1*β* Expression in Peripheral Blood Monocytes

**Figure 2:**
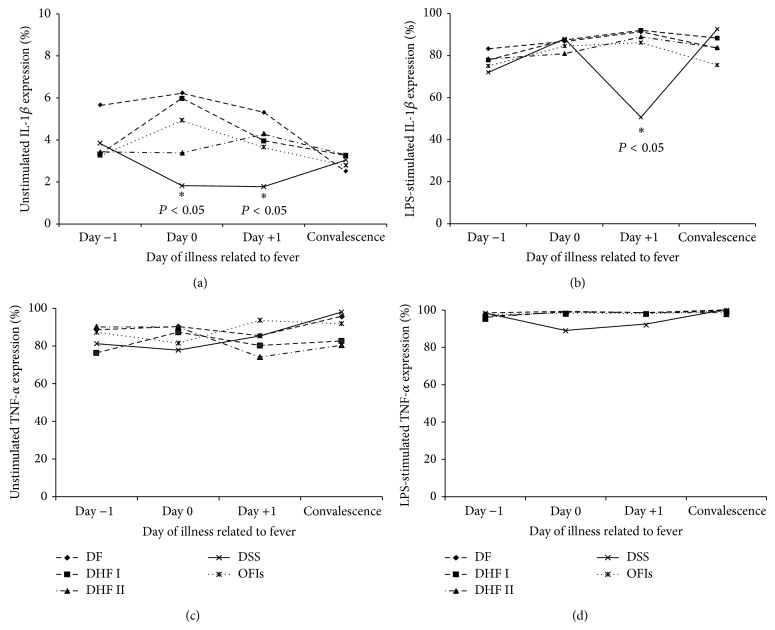
Mean levels of % expression of unstimulated and LPS-stimulated intracellular IL-1*β* ((a) and (b)) and mean levels of % expression of unstimulated and LPS-stimulated intracellular TNF-*α* ((c) and (d)) in monocytes of DF, DHF grades I and II, DSS, and OFIs patients, followed by the day of illness related to fever.

Intracellular TNF-*α* and IL-1*β* expressions in peripheral blood monocytes (CD14+) were determined by flow cytometry and defined as monocyte activation (Figure 2). The percentage of intracellular IL-1*β* expression in DSS patients on Day 0 and Day +1 was significantly lower than in DF, DHF I, DHF II, and OFIs patients (*P* < 0.05) (Figure 2(a)). After LPS-stimulation, monocytes from both dengue and OFIs patients had increased intracellular IL-1*β* expression (Figure 2(b)), except in DSS patients which showed statistically significant decreased levels especially on Day +1 as compared with other groups (*P* < 0.05). For intracellular TNF-*α* expression on unstimulated monocytes, there was no statistical significant difference among the studied groups (Figure 2(c)). However, for LPS-stimulated monocytes, the intracellular TNF-*α* in DSS patients showed slightly decreased levels than other groups on Day 0 and Day +1 (Figure 2(d)).

### 3.5. Surface CD11b Expression on Peripheral Blood Monocytes

**Figure 3:**
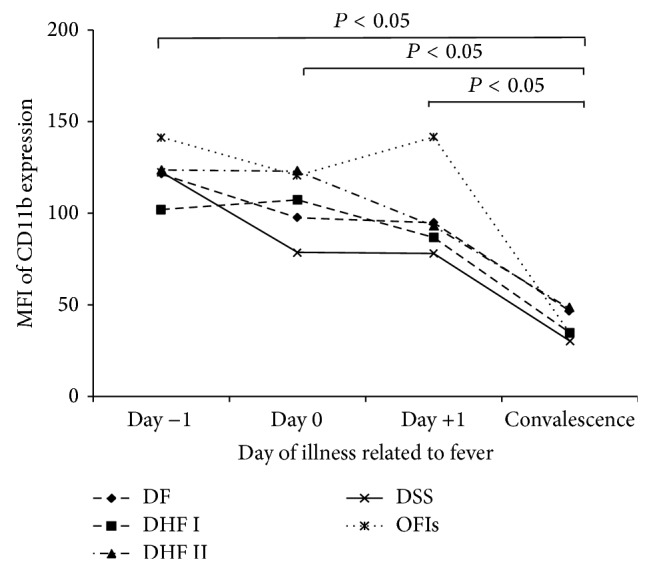
The mean fluorescence intensity (MFI) of surface CD11b expression on the surface of monocytes in DF, DHF grades I and II, DSS, and OFIs patients, followed by the day of illness related to fever (unit as a.u. or arbitrary units of fluorescence).

Surface CD11b fluorescence intensity on monocyte (CD14+) was determined by using flow cytometry and it was measured as an additional marker of monocyte activation. The levels of MFI of surface CD11b were not significantly different among the studied groups. DSS patients had the lowest level of MFI of surface CD11b especially on Day 0 and Day +1 (Figure 3). However, the MFI of surface CD11b in patients infected with dengue virus was significantly higher on Day −1, Day 0, and Day +1 than on the day of convalescence (*P* < 0.05).

### 3.6. Correlation Analysis

**Figure 4:**
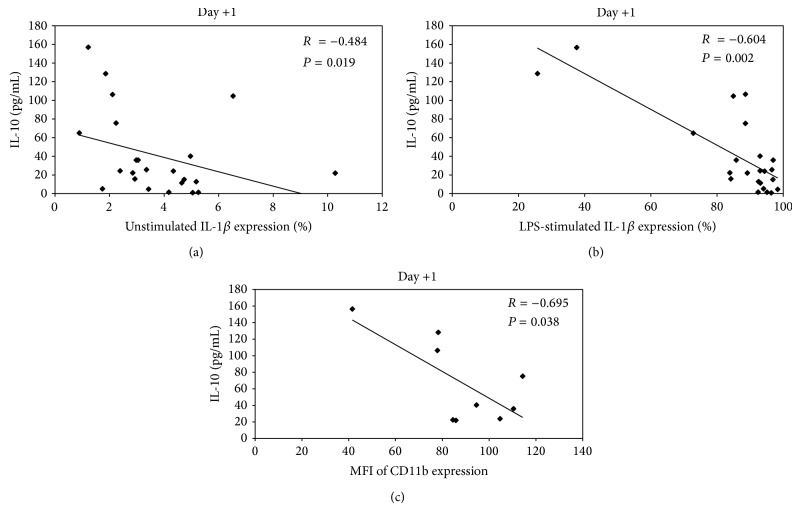
Correlation between IL-10 levels versus unstimulated and LPS-stimulated intracellular IL-1*β* expression ((a) and (b)) and versus MFI of CD11b expression (c) in DHF patients.

The negative correlations between IL-10 levels and unstimulated and LPS-stimulated intracellular IL-1*β* and MFI of CD11b expression were shown in Figure 4 with *r* = −0.484 (*P* = 0.019); *r* = −0.604 (*P* = 0.002) and *r* = −0.695 (*P* = 0.038) for IL-10 versus unstimulated IL-1*β*, versus LPS-stimulated IL-1*β*, and versus MFI of CD11b expression, respectively.

## 4. Discussion

Pathogenesis of DHF has not been completely understood. There are many hypotheses that have been studied to explain the vascular leakage phenomenon in DHF especially in DSS. Vascular leakage occurs when endothelial cells are activated or damaged and are followed by the loss of their barrier function. There are many causes of endothelial cells activation that have been investigated in dengue virus infection such as dengue virus itself, secretion from monocyte/macrophage, complement activation, and cytokine production [9, 31–33]. In this study, monocyte status and various kinds of mediators related to monocytes were investigated in order to understand the mechanism related to vascular leakage and immune responses in patients infected with dengue virus.

Several studies reported that monocytes are proposed to be the primary target cells of dengue virus infection in human [6, 7]. Dengue virus infected cultured human monocytes can increase TNF-*α* expression and apoptosis [34]. This study demonstrated the presentation of dengue viral antigen in monocytes among PBMCs of patients infected with dengue virus. Recently, Durbin et al. [4] characterized the phenotype of PBMCs from patients infected with dengue virus and found that the cells containing dengue antigen expressed the phenotype typical of activated peripheral blood monocytes. The findings in this study confirm the evidence that monocytes are the target cells of dengue viral infection in human peripheral blood. CD11b expression is used to be a marker of monocyte activation* in vivo* and plays an important role in the recruitment of PMNs and monocytes to the sites of infection [34]. Previous studies have shown a higher level of CD11b expression on monocytes in many diseases [35, 36]. Activated monocyte was also found to play a potential role in the activation of vascular endothelium, as well as monocyte adherence which is related to IL-10 secretion [37, 38]. In this study, the monocytes activation was detected as demonstrated by the increased CD11b expression in all types of studied groups as compared to the day of convalescences. Astonishingly, the lowest expression of CD11b on monocytes was detected in DSS patients especially in febrile and toxic stages as previously reported that the IL-10 could suppress CD11b expression [38, 39].

Dengue-infected monocytes could stimulate cytokines/chemokines production (e.g., TNF-*α* and IL-1*β*) which are known to activate endothelial cell. TNF-*α* and IL-1*β* act locally on endothelial cells to increase vascular permeability and adhesion molecules expression, facilitating blood leukocyte adherence and diapedesis [8, 31, 40]. Accordingly, the determination for the expression of intracellular cytokines in dengue-infected monocytes, that is, TNF-*α* and IL-1*β*, as unstimulated and LPS-stimulated conditions in the presence of BFA, which prevented the secretion of any intracellular cytokine that had accumulated in the cytoplasm [41], showed that both circulating unstimulated and LPS-stimulated monocytes from DSS patients reduced the IL-1*β* and TNF-*α* production less than the other groups. These findings are similar to the report of Suharti et al. which has studied patients with DSS and found that the* ex vivo* LPS-stimulated production of the proinflammatory cytokines TNF-*α* and IL-1*β* were considerably depressed but returned to normal on recovery [42]. One possible reason is that the continuous activation of monocytes might reduce the capacity of the cells to produce cytokines in response to physiological agonists then leading to monocyte exhaustion. Another reason of downregulation of intracellular cytokines may result from the action of the anti-inflammatory cytokine IL-10 which may reflect a physiologic counter response to the proinflammatory cytokine production by various mechanisms [43–45]. However, IL-10 has been shown to be increased in DHF patients associated with the degree of plasma leakage quantified by the size of the pleural effusion [18, 19]. Also, IL-10 was found to be associated with reduction of T cells and T cell apoptosis [46]. In addition, this study found the highest level of IL-10 in DSS patients which has negative correlation with the percentages of intracellular IL-1*β* expression in both unstimulated and LPS-stimulated monocytes especially in DHF patients. Furthermore, the negative correlation between IL-10 and MFI of CD11b expression in DHF patients which indicated the monocytes exhaustion was also demonstrated.

In summary, these results render support of enhanced both proinflammatory cytokine TNF-*α* and anti-inflammatory cytokine IL-10 production in accordance with the clinical grading of dengue viral infection. The cause and effect relationship of these cytokines as well as hematological changes and monocyte status regarding the clinical severity may explain the mechanism leading to vascular leakage in severe dengue-infected patients.

## Figures and Tables

**Table 1:** Clinical diagnosis and demographic information of studied patients.

Diagnosis	Number of patients			Age (years)
	Total	Male	Female	Mean (range)
Dengue fever	36	16	20	10.5 (4–14)
DHF grade I	33	16	17	12.0 (5–17)
DHF grade II	27	12	15	12.3 (7–15)
DSS	11	5	6	8.9 (4–15)
OFIs	8	5	3	11.2 (5–19)

Total	115	54	61	11.1 (4–19)

DHF: dengue hemorrhagic fever; DSS: dengue shock syndrome; OFIs: other febrile illnesses.

**Table 2:** The distribution of studied samples followed by the day of illness related to fever.

Diagnosis	Day of illness related to fever						Total
	Day −2	Day −1	Day 0	Day +1	Day +2	Conval	
Dengue fever	7	17	36	30	10	31	131
DHF grade I	7	18	29	33	3	31	121
DHF grade II	2	14	27	23	7	19	92
DSS	2	6	9	11	9	11	48
OFIs	3	6	8	6	4	7	34

Total	21	61	109	103	33	99	426

DHF: dengue hemorrhagic fever; DSS: dengue shock syndrome; OFIs: other febrile illnesses; Conval: convalescence.

**Table 3:** Mean of maximum, minimum, and percentage of increment of Hct in patients with DF, DHF grades I and II, DSS, and OFIs.

	Type				
	DF	DHF I	DHF II	DSS	OFIs
Number of patients	36	33	27	11	8
Maximum Hct (%)	39.5 ± 4.0	43.5 ± 3.9	44.7 ± 4.3	47.9 ± 8.1	35.6 ± 6.1
Minimum Hct (%)	34.6 ± 3.8	36.1 ± 4.0	35.7 ± 4.7	32.8 ± 3.9	31.0 ± 5.9
Mean increment of Hct (%)	14.4	21.1	26.0	37.7	12.8

**Table 4:** Mean ± SD of platelet counts in DF, DHF grades I and II, DSS, and OFIs patients, followed by the day of illness related to fever.

Platelet count (×10^6^/L)						
Type	Day −2	Day −1	Day 0	Day 1	Day 2	Conval^*^
DF (*n*)	115 ± 33 (7)	112 ± 41^**^ (17)	85 ± 34^**^ (36)	86 ± 31^**^ (30)	129 ± 47^¶^ (10)	360 ± 131 (31)
DHF I (*n*)	77 ± 29 (7)	64 ± 32 (18)	61 ± 26 (29)	56 ± 25 (33)	59 ± 44 (3)	376 ± 147 (31)
DHF II (*n*)	65 ± 37 (2)	66 ± 30 (14)	52 ± 26 (27)	57 ± 34 (23)	72 ± 53 (7)	319 ± 99 (19)
DSS (*n*)	80 ± 49 (2)	63 ± 46 (6)	40 ± 20^¥^ (9)	61 ± 44 (11)	51 ± 40 (9)	385 ± 77 (11)
OFIs (*n*)	110 ± 41 (3)	128 ± 15 (6)	147 ± 69^‡^ (8)	126 ± 51 (6)	124 ± 29 (4)	310 ± 66 (7)

Conval^*^ = Convalescence.

DF versus DHF/DSS: ^**^ *P* < 0.05; DSS versus DF, DHF I, and OFIs: ^¥^ *P* < 0.05.

OFIs versus DHF/DSS: ^‡^ *P* < 0.05; DF versus DHF/DSS: ^¶^ *P* < 0.05.

**Table 5:** Mean ± SD of the level of interleukin-10 (IL-10) in patients with DF, DHF grades I and II, DSS, and OFIs patients, followed by the day of illness related to fever.

IL-10 (pg/mL)						
Type	Day −2	Day −1	Day 0	Day +1	Day +2	Conval^*^
DF (*n*)	44.0 ± 28.8 (7)	41.1 ± 22.9^**^ (17)	56.7 ± 37.5 (36)	29.0 ± 14.3 (30)	14.4 ± 10.1 (10)	1.2 ± 0.7 (31)
DHF I (*n*)	19.4 ± 11.6 (7)	93.4 ± 41.8 (18)	55.3 ± 32.8 (29)	19.7 ± 11.5 (33)	8.5 ± 6.3 (3)	1.2 ± 1.0 (31)
DHF II (*n*)	52.3 ± 34.9 (2)	125.6 ± 60.1 (14)	57.8 ± 34.3 (27)	25.3 ± 14.4 (23)	10.2 ± 7.4 (7)	1.1 ± 0.6 (19)
DSS (*n*)	29.1 ± 11.2 (2)	153.6 ± 65.0^¶^ (6)	41.6 ± 25.4 (9)	68.5 ± 40.7^¶^ (11)	16.5 ± 10.7 (9)	1.0 ± 0.4 (13)
OFIs (*n*)	2.9 ± 2.0 (3)	28.5 ± 15.9^¥^ (6)	17.9 ± 10.1^‡^ (8)	8.8 ± 6.6 (6)	7.8 ± 3.6 (4)	0.8 ± 0.2 (7)

Conval^*^ = Convalescence.

DF versus DHF/DSS: ^**^ *P* < 0.01; OFIs versus DHF/DSS: ^¥^ *P* < 0.05.

OFIs versus dengue (DF + DHF): ^‡^ *P* < 0.05; DSS versus DF and OFIs: ^¶^ *P* < 0.05.

**Table 6:** Mean ± SD of the level of tumor necrosis factor-alpha (TNF-*α*) in patients with DF, DHF grades I and II, DSS, and OFIs patients, followed by the day of illness related to fever.

TNF-*α* (pg/mL)						
Type	Day −2	Day −1	Day 0	Day +1	Day +2	Conval^*^
DF (*n*)	4.0 ± 2.0 (7)	4.1 ± 2.0 (17)	4.3 ± 1.9^¥^ (36)	3.8 ± 1.5 (30)	3.5 ± 1.2 (10)	2.3 ± 0.9 (31)
DHF I (*n*)	4.2 ± 1.3 (7)	4.4 ± 1.6 (18)	3.3 ± 1.5 (29)	3.1 ± 1.0 (33)	2.2 ± 0.1 (3)	2.3 ± 1.2 (31)
DHF II (*n*)	5.7 ± 2.4 (2)	5.1 ± 1.5 (14)	3.8 ± 1.4 (27)	3.5 ± 1.6 (23)	3.0 ± 1.1 (7)	2.1 ± 0.7 (19)
DSS (*n*)	4.1 ± 1.7 (2)	4.1 ± 2.5 (6)	2.8 ± 1.8^‡^ (9)	4.8 ± 2.6^¶^ (11)	3.4 ± 1.3 (9)	2.4 ± 0.8 (13)
OFIs (*n*)	2.7 ± 2.0 (3)	3.7 ± 1.1^**^ (6)	3.2 ± 1.6 (8)	4.2 ± 3.1 (6)	4.7 ± 2.7^‡‡^ (4)	2.3 ± 0.4 (7)

Conval^*^ = convalescence.

DHF II versus OFIs: ^**^ *P* < 0.05; DF versus DHF I and DSS: ^¥^ *P* < 0.05.

DSS versus DF and DHF II: ^‡^ *P* < 0.05; DSS versus DHF I: ^¶^ *P* < 0.05.

DHF I versus OFIs: ^‡‡^ *P* < 0.05.

**Table 7:** Mean ± SD of the level of interleukin-1 beta (IL-1*β*) in patients with DF, DHF grades I and II, DSS, and OFIs patients, followed by the day of illness related to fever.

IL-1*β* (pg/mL)						
Type	Day −2	Day −1	Day 0	Day +1	Day +2	Conval^*^
DF (*n*)	0.5 ± 0.2 (7)	2.7 ± 2.7 (17)	2.1 ± 1.7 (36)	2.4 ± 1.2 (30)	2.1 ± 1.5 (10)	1.2 ± 1.3 (31)
DHF I (*n*)	1.0 ± 0.7 (7)	1.0 ± 0.9 (18)	1.1 ± 1.1 (29)	1.3 ± 1.4 (33)	0.4 ± 0.2 (3)	1.0 ± 1.2 (31)
DHF II (*n*)	0.5 ± 0.1 (2)	0.8 ± 1.1 (14)	1.0 ± 1.1 (27)	0.9 ± 1.1 (23)	0.8 ± 0.7 (7)	1.0 ± 1.1 (19)
DSS (*n*)	0.6 ± 0.01 (2)	1.4 ± 0.7 (6)	1.0 ± 0.9 (9)	0.9 ± 0.7 (11)	0.8 ± 0.4 (9)	1.2 ± 0.7 (13)
OFIs (*n*)	0.8 ± 0.2 (3)	1.9 ± 3.0 (6)	1.9 ± 1.9 (8)	2.2 ± 2.4 (6)	1.4 ± 1.1 (4)	0.9 ± 0.4 (7)

Conval^*^ = Convalescence.
